# Development of Carcinoembryonic Antigen Rapid Detection System Based on Platinum Microelectrode

**DOI:** 10.3389/fchem.2022.899276

**Published:** 2022-06-20

**Authors:** Jiali Zhai, Piyou Ji, Yu Xin, Yifan Liu, Qianwen Qu, Wentong Han, Guangtao Zhao

**Affiliations:** ^1^ School of Rehabilitation Medicine of Binzhou Medical University, Yantai, China; ^2^ Yantai Affiliated Hospital of Binzhou Medical University, Yantai, China; ^3^ School of Medical Imaging, Binzhou Medical University, Yantai, China; ^4^ School of Basic Medicine, Binzhou Medical University, Yantai, China

**Keywords:** tumor markers, platinum microelectrode, carcinoembryonic antigen, aptamer, square wave voltammetry

## Abstract

Rapid and highly sensitive detection of carcinoembryonic antigen (CEA) in blood could effectively improve the diagnostic sensitivity of colorectal cancer. In this work, a platinum microelectrode (PtμE) modified with gold nanoparticles was developed as a microsensor for the detection of CEA. As the recognition element, a CEA aptamer modified with sulfhydryl could be conjugated onto the surface of the PtμEs/Au. The quantitative analysis of the concentration of CEA [CEA] by the prepared PtμEs/Au aptasensor was carried out through square wave voltammetry. Under the optimized conditions, the PtμEs/Au aptasensor exhibits a linear response toward [CEA] in the range of 1.0 × 10^–11^—1.0 × 10^–7^ g/ml (S = 5.5 nA/dec, *R* ^2^ = 0.999), and the detection limit is 7.7 × 10^–12^ g/ml. The PtμEs/Au aptasensor also has good selectivity against other types of proteins existing in blood. The availability of the developed assay toward [CEA] in blood samples was investigated, and the results agreed well with those obtained through electrochemiluminescence provided by the hospital, and the volume of the blood sample for detection is only 20 μl. Herein, the proposed detection system could be used for the quantitative analysis of CEA in blood, with the advantages of high sensitivity, short time, and low cost. Moreover, the PtμEs/Au aptasensor has a potential application in clinical diagnosis.

## 1 Introduction

Cancer is one of the most serious threats to our life, and its mortality rate could be greatly reduced by the improvement of the clinical diagnosis of cancers at an early stage (Chinen et al., 2015). The level of the tumor markers in serum, tissue, urine, or saliva is an important indicator of the existence and growth of cancers. Therefore, the detection of tumor markers with high sensitivity and specificity remains the long-term goal of clinical diagnosis (Qi et al., 2020; Tang et al., 2020).

Nowadays, various methods have been developed for the detection of tumor markers, and most of them are based on immunoassays, such as enzyme-linked immunosorbent assay (Yen et al., 2020), fluorescence (Li et al., 2011), and electrochemical immunosensor (Chen et al., 2013). Despite the advantages of good specificity and sensitivity, these methods also suffered from the problems of tedious preparation and expensive antibodies. The proteomic techniques based on two-dimensional electrophoresis (Hodgkinson et al., 2012) and mass spectrometry (Chen et al., 2012) could also realize the detection of tumor markers with high accuracy, multiplexed quantitation, automation, and miniaturization. However, the expensive instruments and high requirement for operation skills limit their further application. Moreover, molecular biotechnology including polymerase chain reaction (Koike et al., 2004), and fluorescence *in situ* hybridization (Lv et al., 2016) are good analytical strategies for the detection of the tumor markers, while these methods have the drawbacks of high cost and are time consuming.

The electrochemical sensors have been widely used in clinical diagnosis (Ng et al., 2010), environmental analysis (Chumbimuni-Torres et al., 2008; Ummadi et al., 2016), and biological research (Hao et al., 2016; Zhang et al., 2019), with attractive properties of simple operation, portability, convenience, and continuous rapid detection. Moreover, the electrochemical methods based on square wave voltammetry arouse the interest of scientists for their features of high sensitivity, low cost, fast response, and easy miniaturization (Chiavassa and La-Scalea, 2018; Frkonja-Kuczin et al., 2020). Therefore, the electrochemical sensors based on square wave voltammetry (SWV) could be a potential tool for the detection of tumor markers with high sensitivity, especially the electrochemical microsensors, which have been widely applied in the food inspection (Fysun et al., 2020) and life science (Taylor et al., 2019; Gładysz and Skibiński, 2020).

The aptamers are single-stranded nucleic acids synthesized as the capturing agent for their cognate targets due to their high affinity and selectivity characteristics (Azadbakht et al., 2016; Citartan et al., 2016). Compared with antibodies, aptamers have the unique features of low cost, easy synthesis, and a wide range of target molecules, including protein, amino acids, small molecules, and even cells (Taghdisi et al., 2016). However, the research using the microelectrode as a microsensor combined with the aptamer as recognition element toward tumor markers is rather rare.

In this work, a platinum microelectrode (PtμE) was developed as a microsensor for the detection of tumor markers in blood. The aptamer modified with sulfhydryl was used as the recognition element, and it could be immobilized onto the surface of the PtμE with the electrodeposition of gold nanoparticles. Taking carcinoembryonic antigen (CEA) as a model, which is an important indicator of the state of colorectal cancers with a cutoff value of less than 5 ng/ml in serum (Chen et al., 2018; Tang et al., 2020), the PtμEs/Au aptasensor was proposed using for the clinical measurement of CEA in the blood through SWV with high sensitivity and selectivity. The experimental conditions of the detection assay have also been optimized.

## 2 Experimental Section

### 2.1 Chemicals

Bovine serum albumin (BSA), trypsin, PBS (pH 7.2–7.4, 136.89 mM NaCl, 2.67 mM KCl, 8.24 mM Na_2_HPO_4_, 1.76 mM NaH_2_PO_4_), and sulfhydryl-modified CEA aptamer HS-C6-AAAAAAATACCAGCTTATTCAATT (Tang et al., 2020) were purchased from Shanghai Sangon Biotech Co., Ltd. (Shanghai, China). Human IgG was purchased from Beyotime Biotechnology. The CEA protein and alpha-fetoprotein (AFP) protein were purchased from Fitzgerald Inc. Chloroauric acid (HAuCl_4_) was purchased from Macklin Biotech Co., Ltd. (Shanghai, China). Milli-Q ultrapure water (18.2 MΩ cm specific resistance) was used throughout. All the other chemicals were of analytical reagent grade.

### 2.2 Fabrication of the Platinum Microelectrode

**FIGURE 1:**
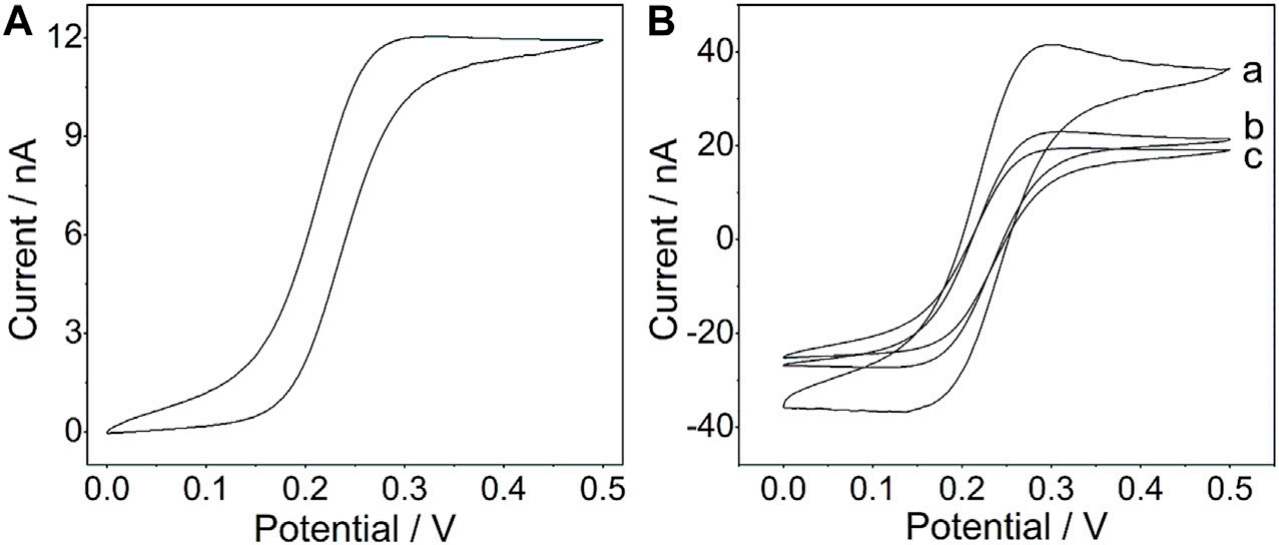
**(A)** Cyclic voltammogram of the PtμE in a 4 mM K_4_ [Fe(CN)_6_] solution (1 M KCl, pH 7.0); **(B)** Cyclic voltammograms of the PtμE/Au electrodes (a) PtμE/Au, (b) bare PtμE, and (c) PtμE/Au aptasensor in 0.1 M KCl solution. The scan rate was 50 mV s^−1^.

A platinum wire with a diameter of 21.3 μm (Conghang Co., Ltd., Shanghai, China, 99.9%) was used to fabricate the platinum microelectrode, which is denoted as PtμE, and the procedure is similar to that in the previous report (Zhao et al., 2019). The prepared PtμE electrodes were left in 1.0 M HNO_3_ for 15 min and then were cleaned ultrasonically in deionized water and ethanol for 5 min. As shown in Figure 1A, the voltammetric characteristic of the PtμE shows a sigmoid-shaped voltammogram, which is the typical characteristic of the microelectrode (Gyurcsányi et al., 1998).

### 2.3 Fabrication of the Aptasensors

The gold nanoparticles were electrodeposited onto the surface of PtμE through galvanostatic electrochemical polymerization in an aqueous solution of 1 μM HAuCl_4_ under a constant current of 50 nA for 50, 100, 200, and 300 s to produce a total polymerization charges of 2.5, 5, 10, and 15 μC, respectively. The microelectrodes modified with gold nanoparticles were denoted as the PtμE/Au electrodes (Ihalainen et al., 2011; Hupa et al., 2015). The polymerization was carried out in a three-electrode cell using a Pt wire as the counter electrode, an Ag/AgCl/3 M KCl microelectrode as the reference electrode, and the above-prepared PtμE/Au electrodes as the working electrode. After electrodeposition, the PtμE/Au electrodes were rinsed with deionized water and allowed to dry in air for 1 day.

Proper folding of the CEA aptamer was obtained by heating at 95°C for 5 min and then annealing immediately on ice for 15 min. After being incubated with 20 μl CEA aptamer (1 μM) in a 0.2-ml centrifuge tube for 1 h at room temperature, the PtμE/Au electrodes were rinsed with PBS buffer to remove the nonspecific absorbed CEA aptamer. The PtμE/Au electrodes immobilized with CEA aptamer were denoted as PtμEs/Au aptasensor. The incubation conditions were also optimized to achieve a high signal.

### 2.4 Apparatus and Measurements

SWV was used to characterize each step of the PtμEs/Au aptasensor fabrication using a CHI 660E electrochemical workstation (Shanghai Chenhua Apparatus Corporation, China). SWV was performed from −0.1 to 0.5 V in a 5.0 mM [Fe(CN)_6_]^4-/3-^ solution containing 0.1 M KCl, the amplitude was 50 mV, step potential was 5 mV, and the frequency was 25 Hz. Cyclic voltammetry (CV) was carried out in 0.1 M KCl solution. The SWV and CV measurements were both performed using a three-electrode system, comprising the PtμE or PtμE/Au electrode as the working electrode, the Ag/AgCl/3 M KCl microelectrode as the reference electrode, and a Pt wire as the counter electrode.

### 2.5 Analytical Application

In order to investigate the availability of the prepared PtμEs/Au aptasensors in clinical diagnosis, the blood samples were collected from in-patients of Yantai Affiliated Hospital of Binzhou Medical University for analysis. The fabricated PtμEs/Au aptasensors were dipped into 20 μl of each blood sample in a 0.2-ml centrifuge tube without pretreatment. After being incubated with each blood sample for 1 h at room temperature, the PtμEs/Au aptasensors were washed thoroughly with PBS solution for SWV measurements, and the values of the concentration of CEA [CEA] were calculated through the plotting linear curve of net current change (∆I) between the peak current of the PtμEs/Au aptasensors without CEA versus each [CEA] (Gui et al., 2018; Rizwan et al., 2018). For comparison, the [CEA] in blood samples was also measured in the Laboratory Center of the Yantai Affiliated Hospital of Binzhou Medical University through the electrochemiluminescence method.

## 3 Results and Discussion

**SCHEME 1:**
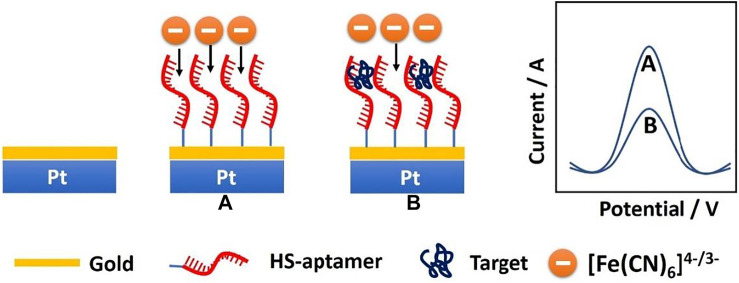
Schematic illustration of the PtμEs/Au aptasensor for quantitative analysis of CEA through square wave voltammetry in blood.

The fabrication scheme of the CEA microsensor is indicated in Scheme 1. When the CEA aptamer is bound onto the surface of the PtμEs/Au, the peak current of the SWV would decrease due to the decrease of the active area of the PtμEs/Au, and the peak current of the SWV would further decrease when the CEA is captured by the PtμEs/Au aptasensor through the special recognition of the CEA aptamer, which is caused by the inhibition of the electron transfer of the redox molecule [(Fe(CN)_6_)^4-/3-^] to the surface of the PtμEs/Au (Hyun et al., 2016; Mahshid et al., 2019). The net current change (∆I) between the peak current of the PtμEs/Au aptasensor recorded at ca. 0.23 V before and after incubation with CEA can be used for the quantification analysis of [CEA].

### 3.1 Cyclic Voltammogram Measurements

The CV was used to investigate the redox capacitance of the microelectrodes before and after electrodeposition of the gold nanoparticles (Figure 1B). The interfacial capacitance of the PtμEs/Au could be calculated by summing the charge current in the positive and negative scan directions and dividing the sum by twice the scan rate. As shown in Fig. S1, the capacitance of the PtμEs/Au is calculated to be 78.3 nF cm^−2^, which is much higher than that of the bare PtμE electrodes (41.3 nF cm^−2^) (Zheng et al., 2009) The capacitive current of the PtμE/Au electrode is much higher than that of the bare PtμE electrodes, which reveals that the redox capacitance of the microelectrodes is enhanced due to the presence of a gold nanoparticle film. Moreover, according to the Randles–Sevcik equation: i_p_ = 2.69 × 10^5^ n^3/2^AD^1/2^ V^1/2^C_0_, where i_p_ is the peak current (A), n is the number of electrons, A is the electrode area, D is the diffusion coefficient 6.7 × 10^–6^ (cm^2^ S^−1^), V is the scan rate (V s^−1^), and C_0_ is the concentration (mol cm^−3^), and the surface area A of the PtμEs, PtμEs/Au, and PtμEs/Au aptasensor can be determined (Rizwan et al., 2018). It is found that the PtμEs/Au possessed about 116% more surface area than the bare PtμEs and about 183% higher than the PtμEs/Au aptasensor, and the electronic conductivity is decreased obviously due to the immobilization of the CEA aptamer. Therefore, the capacitive current of the PtμEs/Au decreases after the immobilization of the CEA aptamer, which results from the decrease of the surface area A of the electrodes.

### 3.2 Electrodeposition of the Gold Nanoparticles

**FIGURE 2:**
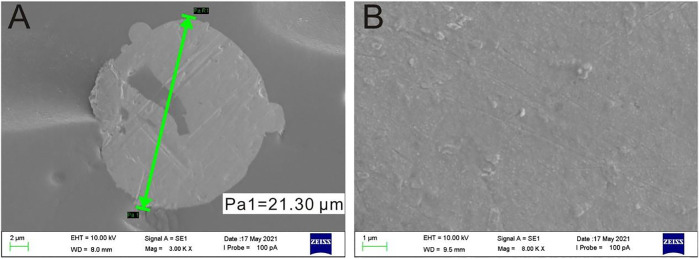
SEM images of the bare PtμE **(A)** and gold nanoparticle film covered onto the surface of the PtμE **(B)**.

The gold nanoparticles electrodeposited onto the surface of PtμE could not only act as solid contact which would improve the electrochemical property of the PtμE but could also make the CEA aptamer modified with sulfhydryl conjugated onto the surface of the PtμE directly. The SEM images revealed that the PtμE electrode has a smooth surface with a diameter of 21.3 μm (Figure 2A), while the PtμE/Au electrode has a rough and compact morphology (Figure 2B). The thickness of the gold nanoparticle layer could be reflected through the capacitive current of the cyclic voltammograms, which can be well-controlled by the amount of the polymerization charge from 2.5 to 15 μC. As shown in Supplementary Figure S1, the capacitive current of the bare microelectrode is less than 20 nA, while the capacitive current increases with the increase in the deposited polymerization charge, and the capacitive current is more than 40 nA when the polymerization charge reaches 10 μC. Therefore, the redox capacitance of the electrodes is enhanced obviously due to the modification of the gold nanoparticles, while the capacitive current of the electrodes no longer increases obviously even if the polymerization charge is up to 15 μC (Crespo et al., 2009).

### 3.3 Optimization of the Experimental Conditions

**FIGURE 3:**
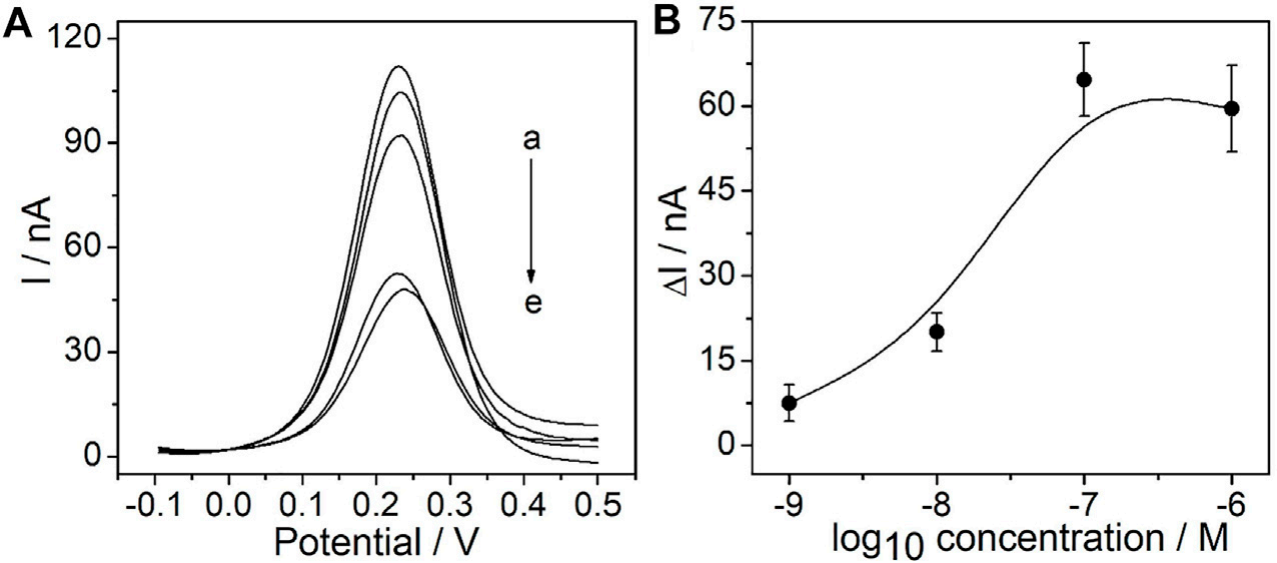
Electrochemical signal and calibration plot of the PtμE/Au aptasensors: **(A)** SWV curve of the PtμE/Au electrodes incubated with CEA aptamers, (a) without the CEA aptamer, (b) 10^–9^ M, (c) 10^–8^ M, (d) 10^–6^ M, and (e) 10^–7^ M; **(B)** SWV calibration plot of PtμE/Au incubated with the CEA aptamer range from 10^–9^ M to 10^–6^ M.

As the recognition element, the CEA aptamer superstructure could be assembled onto the surface of the PtμEs/Au electrodes to form the electrochemical CEA aptasensor (Jiang et al., 2019). In order to obtain the optimal response of the experiment, the concentration of the CEA aptamer used for the preparation of the PtμE/Au aptasensor was optimized. As shown in Figure 3A the SWV response of the PtμE/Au was recorded after being incubated with various concentrations of the CEA aptamer from 10^–9^ M to 10^–6^ M. The SWV peak current decreases with the increase of the concentration of the CEA aptamer, while the peak current no longer decreases when the CEA aptamer concentration is up to 10^–7^ M. Therefore, the 10^–7^ M CEA aptamer was selected for further assay (Figure 3B).

**FIGURE 4:**
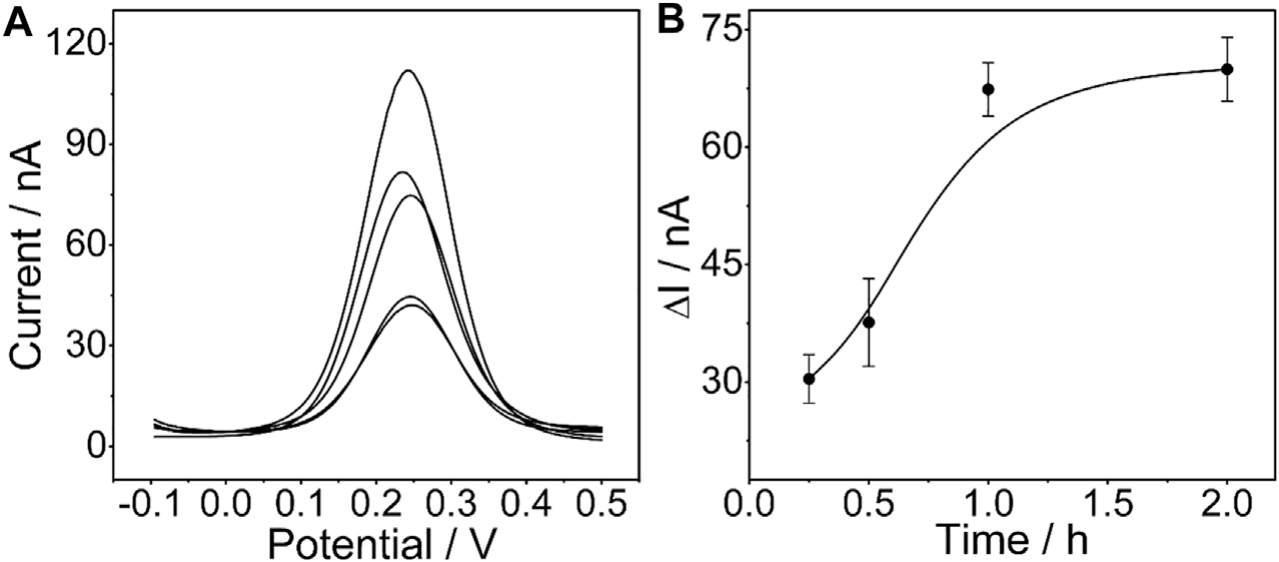
**(A)** SWV curve of the PtμE/Au electrodes incubated with 10^–7^ M CEA aptamers for (a) without the CEA aptamer, (b) 0.25 h, (c) 0.5 h, (d) 1 h, and (e) 2 h; **(B)** SWV calibration plot of PtμE/Au electrodes incubated with 10^–7^ M CEA aptamer for a different time from 0.25 to 2 h.

The influence of the incubation time of the determined CEA aptamer and PtμE/Au electrodes was also investigated. The results show that the peak current of the SWV curve decreases with the increase of the incubation time of the PtμE/Au electrodes incubated with the 10^–7^ M CEA aptamer from 0.25–2 h, and it would no longer decrease when the incubation time is up to 1 h (Figure 4). Therefore, the incubation time of 1 h was used for further study.

The influence of the deposited polymerization charge of the gold nanoparticles onto the surface of the PtμEs on the SWV performance of the PtμEs/Au aptasensor was investigated. The PtμEs modified with gold nanoparticles with different deposited polymerization charges from 2.5 to 15 μC were incubated with the 10^–7^ M CEA aptamer for 1 h, and then the peak current of the SWV response was recorded. As shown in Supplementary Figure S2, the SWV peak current decreases with the increase of the polymerization charge of the gold nanoparticles, while the peak current almost stays the same when the polymerization charge ranges from 5 to 15 μC. Taking the redox capacitance of the PtμEs/Au and the SWV performance of the PtμEs/Au aptasensor into account, the polymerization charge of 10 μC was used for further assay.

### 3.4 Sensitivity, Selectivity, and Reproductivity of the PtμE/Au Aptasensors

**FIGURE 5:**
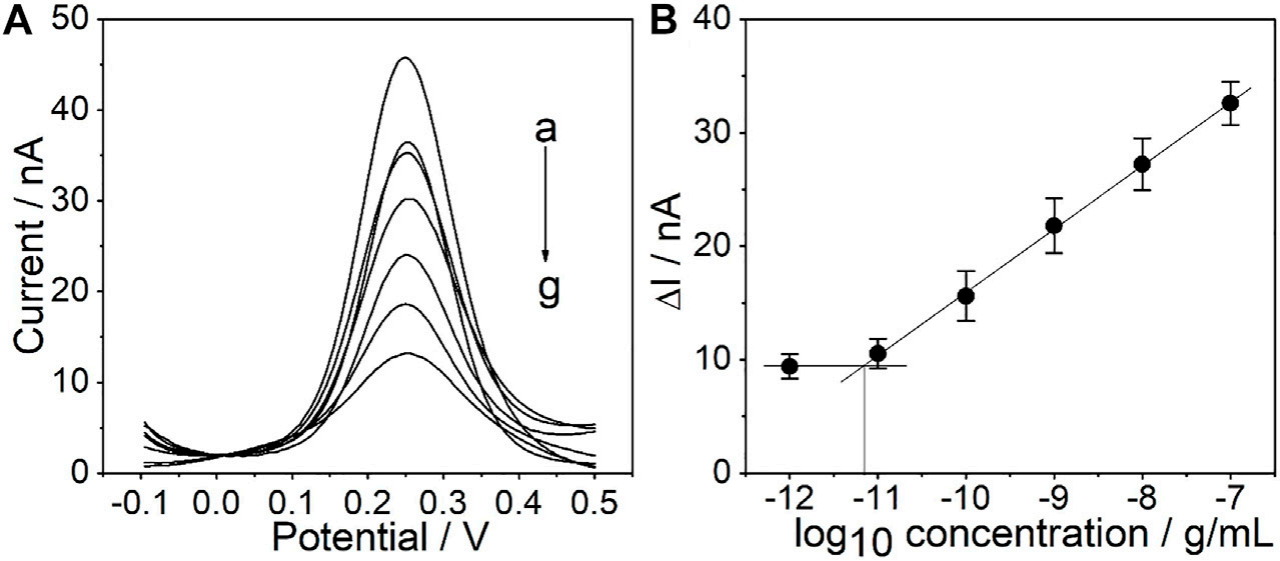
**(A)** SWV curve of the PtμE/Au aptasensors for CEA, (a) without CEA, (b) 10^–12^ g/ml, (c)10^–11^ g/ml, (d) 10^–10^ g/ml, (e) 10^–9^ g/ml, (f) 10^–8^ g/ml, and (g) 10^–7^ g/ml; **(B)** SWV calibration plot of PtμE/Au aptasensors recorded for [CEA] range from 10^–12^ g ml^−1^ to 10^–7^ μg ml^−1^.

Under the optimized conditions mentioned above, the sensitivity of the PtμE/Au aptasensor against CEA was investigated by measuring SWV starting at the concentration of 1.0 × 10^–7^ g/ml and diluting the CEA solution by a factor of 10 each time through PBS solution until a limit of detection (LOD) could be detected, and SWVs were recorded in each concentration three times (Taylor et al., 2019; Hannah et al., 2020). A linear relationship between the ∆I and each [CEA] was observed (Caviglia et al., 2020). The PtμEs/Au aptasensor exhibits a linear response toward CEA in the concentration range of 10^–11^-10^–7^ g/ml (S = 5.5 nA/dec, *R* ^2^ = 0.999), and the LOD is 7.7 × 10^–12^ g/ml, which is calculated according to LOD = 3σ/b, where *σ* is the standard deviation of “*n*”, the number of SWV in blank solution, and b represents the slope of the calibration plot (Figure 5) (Shah et al., 2019; Gupta et al., 2020).

**FIGURE 6:**
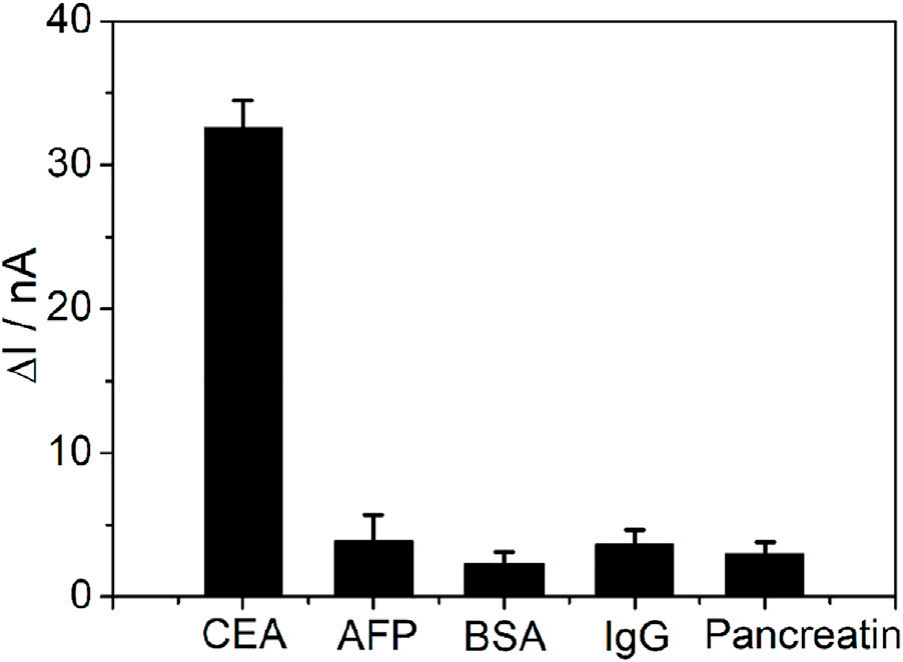
SWV calibration plot of PtμE/Au aptasensors recorded for 0.1 μg ml^−1^ CEA, 1 μg ml^−1^ AFP, 1 μg ml^−1^ BSA, 1 μg ml^−1^ pancreatin, and 1 μg ml^−1^ human IgG.

The selectivity of the PtμE/Au aptasensor was also investigated (Figure 6), and the PtμE/Au aptasensor can selectively distinguish between CEA and other interfering compounds with similar protein structures existing in the blood, such as AFP, BSA, pancreatin, and human IgG, even if the concentration of these proteins was ten times higher than that of the CEA (1.0 μg/ml vs. 0.1 μg/ml). As reproductivity is one of the major concerns of the sensing devices, five freshly prepared PtμEs/Au aptasensors were used for SWV measurement of CEA at the concentration of 0.1 μg/ml, and the standard deviation is 5.1% (Rizwan et al., 2018). Herein, the PtμEs/Au aptasensors have good reproductivity.

### 3.5 Real Sample Analysis

In order to investigate the feasibility of the designed detection assay in clinical applications, the prepared PtμE/Au aptasensor was used for the CEA measurement of the blood samples. As shown in Table 1, the results agree well with those obtained from the electrochemiluminescence measurements, which indicates that the PtμE/Au aptasensor is available for CEA detection in real blood samples.

**TABLE 1:** [CEA] in the blood samples was measured using the developed assay and the electrochemiluminescence measurements.

Samples	Developed detection assay (ng/ml)	Electrochemiluminescence measurements (ng/ml)
Sample 1	7.43 ± 1.37	6.66
Sample 2	2.26 ± 1.58	1.10
Sample 3	5.86 ± 1.43	6.55
Sample 4	0.96 ± 0.34	1.11
Sample 5	30.2 ± 1.98	38.4

## 4 Conclusion

In this work, a highly sensitive and rapid detection system based on the PtμE/Au aptasensor through SWV has been fabricated for the detection of CEA. The PtμE/Au aptasensor was developed using PtμE modified with gold nanoparticles as a microsensor combined with the CEA aptamer as the recognition element. The prepared detection assay can be used for the clinical analysis of CEA in blood samples without pretreatment steps in limited volumes. The detection protocol could be finished within 60 min, and the developed CEA detection assay has good prospects in clinical analysis.

## Data Availability

The original contributions presented in the study are included in the article/Supplementary Materials; further inquiries can be directed to the corresponding author.
