# Supplementary material for: Development of Carcinoembryonic Antigen Rapid Detection System Based on Platinum Microelectrode
Source: Front Chem. 2022 Jun 20;10:899276. doi: 10.3389/fchem.2022.899276 (PMC9252266; doi:10.3389/fchem.2022.899276)
Supplement: Supplementary file 1 [file DataSheet1.PDF]

Development of carcinoembryonic antigen rapid detection system based on platinum microelectrode

Jiali Zhai[a], Piyou Ji[b], Yu Xin[c], Yifan Liu[c], Qianwen Qu[c], Wentong Han[c], and Guangtao Zhao[d*],

[a] *School of Rehabilitation medicine of Binzhou Medical University, Yantai, P.R. 264003, China;*

[b] *Yantai Affiliated Hospital of Binzhou Medical University, Yantai, P.R. 264100, China;*

[c] *School of Medical Imaging, Binzhou Medical University, Yantai, P.R. 264003, China;*

[d] *School of Basic Medicine, Binzhou Medical University, Yantai, P.R. 264003, China;*

Corresponding author. Tel.: +86 535 6913213; Fax: +86 535 6913246.

E-mail address: gtzhao@bzmc.edu.cn (G. Zhao).

Fig. S1

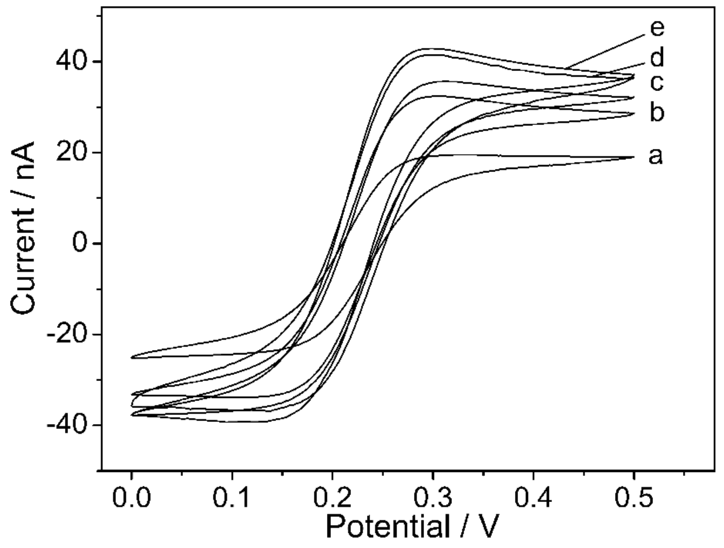

Fig. S1 Cyclic voltammograms of the PtμEs in a 0.1 M KCl solution after modification to obtain the gold nanoparticles films with polymerization charge of (a) 0, (b) 2.5, (c) 5.0, (d) 10.0, and (e) 15.0 μC. The scan rate was 50 mV s^{-1}.

Fig. S2

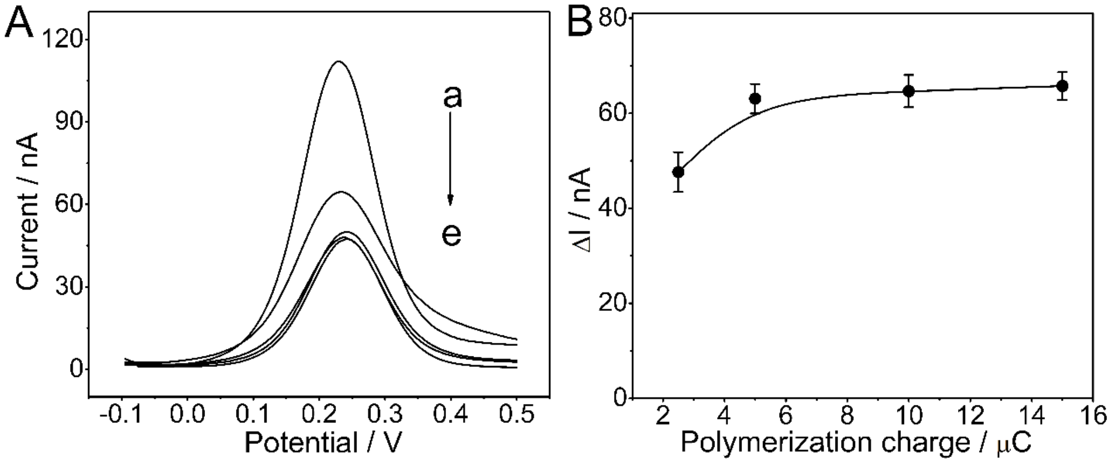

Fig. S2 SWV curve of the PtμE electrodes after the modification of gold nanoparticles film for polymerization charge incubated with 10^{-7} M CEA aptamers (a) without CEA aptamer, (b) 2.5 μC, (c) 5 μC, (d) 10 μC, and (d) 15 μC; (B) SWV calibration plot of PtμE/Au electrodes incubated with 10^{-7} M CEA aptamer with the modification of gold nanoparticles film for polymerization charge from 2.5 μC to 15 μC.
